# Point-of-Care Detection of Salivary Nitrite Based on the Surface Plasmon-Assisted Catalytic Coupling Reaction of Aromatic Amines

**DOI:** 10.3390/bios11070223

**Published:** 2021-07-05

**Authors:** Chen Zhao, Ruyi Shi, Jiale Wu, Xuan Luo, Xiangjiang Liu

**Affiliations:** 1 College of Biosystems Engineering and Food Science, Zhejiang University, Hangzhou 310058, China; zhaochenzc@zju.edu.cn (C.Z.); rucyzd@163.com (R.S.); alexander_wjl@163.com (J.W.); 0016712@zju.edu.cn (X.L.); 2 College of Mechanical Engineering, Xinjiang Agricultural University, Urumqi 830052, China

**Keywords:** surface plasmon-assisted catalysis, surface-enhanced Raman scattering, nitrite, saliva

## Abstract

Rapid quantification of nitrite (NO_2_^−^) in food, drink and body fluids is of significant importance for both food safety and point-of-care (POA) applications. However, conventional nitrite analytical methods are complicated, constrained to sample content, and time-consuming. Inspired by a nitrite-triggered surface plasmon-assisted catalysis (SPAC) reaction, a rapid point-of-care detection salivary nitrate was developed in this work. NO_2_^−^ ions can trigger the rapid conversion of *p*-aminothiophenol (PATP) to *p*,*p*′-dimercaptozaobenzene (DMAB) on gold nanoparticles (GNPs) under light illumination, and the emerged new bands at ca. 1140, 1390, 1432 cm^−1^ originating from DMAB can be used to the quantification of nitrite. Meanwhile, to make the method entirely suitable for on-site fast screen or point-of-care application, the technique is needed to be further optimized. The calibration graph for nitrates was linear in the range of 1–100 µM with a correlation coefficient of 0.9579. The limit of detection was 1 µM. The facile method could lead to a further understanding of the progression and treatment of periodontitis and to guide professionals in planning on-site campaigns to effectively control periodontal diseases.

## 1. Introduction

Nitrite (NO_2_^−^) has been widely used as a food preservative or a chemical fertilizer in agriculture. Nitrite can also accumulate in foods during long-term storage, generated by the nitrogen metabolism processes of bacteria. As a widely existing substance, its possible health implications have been extensively studied. The results have demonstrated that excessive NO^2−^ intake can have serious consequences for physiological systems, such as spontaneous abortions, methemoglobinemia, esophageal cancer, and congenital defects of the central nervous system [1]. The World Heath Organization (WHO) suggests that the nitrite concentrention in drinking water must be kept under 3.0 ppm (65.1 µM) [2] and the guideline value for nitrite proposed by U.S. Environmental Protection Agency (EPA) in drinking water is limited to 1.0 ppm (21.7 µM) [3]. In addition, NO_2_^−^ can also be used as a health biomarker for the human body [4]. Salivary nitrite is reduced by oral bacteria associated with the clinical symptoms of periodontitis (gingival redness, swelling, and bleeding) and can be used to trace oral diseases such as periodontitis [5,6]. Nitrite in urine can be used as an indicator of urinary tract infections [7]. Therefore, rapid on-situ detection of trace-level NO_2_^−^ in foods, drinks, or body fluids is of great necessity for both food safety and point-of-care (POA) applications.

Recently, a variety of analytical methods have been developed to quantify nitrite, such as colorimetry [8], ion chromatography [9,10], flow injection analysis (FIA) [11], capillary electrophoresis [12], and electrochemical techniques [13]. They have been extensively used, but these methods share common drawbacks that include the need for complex sample pretreatment approaches, large reagent and sample volumes, and long detection times, and therefore, they may not be suitable for point-of-care (POA) assays. Thus, a more reliable but simpler and low-cost method for on-site and real-time nitrite ion detection is highly required.

Surface-enhanced Raman scattering (SERS) can strengthen the weak Raman signal by 10^5^~10^8^ when the analyte is close to a nanoscale plasmonic metallic surface (e.g., silver or gold) [14,15], and is capable of detecting a single molecule with high specificity and holds vast potential in numerous fields. Compared with the well-established modern analytical instrumental techniques (such as HPLC, GC-MS, etc.), SERS can avoid complicated and time-consuming sample pretreatment, therefore being more preferred in rapid field screening uses. Examples includes the detection of pesticides [16,17,18], illegal food additives [19,20], veterinary drugs [21,22] and toxins [23,24] in foods. Meanwhile, with the rapid development of miniaturized Raman spectrometers, such as smartphone-based Raman spectrometers, various on-site SERS applications have been developed [25]. Such devices provide inexpensive, user-friendly, and compact tools for point-of-care applications and greatly shorten the distance between laboratory research and real applications.

On other hand, gold nanoparticles (GNPs) have drawn increasing attention as heterogeneous catalysis in the past two decades [26,27]. In-situ monitoring of heterogeneously catalyzed reactions remains challenging because the interface of solution-catalysts is hard to perceive by conventional tools. However, SERS is capable of in-situ monitoring of heterogeneously catalyzed reactions on GNPs [28,29], since GNPs are also SERS-active. SERS combines the advantages of high chemical specificity, high sensitivity, and surface selectivity can provide detailed structural information and binding nature of the molecules and allow detection of analytes at extremely low concentrations. Further, after the SERS experimental investigations, the density functional theory (DFT) quantum chemical calculations serve as complementary has exerted a profound influence on our understanding of the molecular behaviors at the interfaces [30].

In this work, we described a novel POC method for quantification of salivary nitrite based on surface plasmon-assisted catalysis (SPAC) reaction on GNPs. In particular, NO_2_^−^ ions can trigger the rapid conversion of *p*-aminothiophenol (PATP) to *p*,*p*′-dimercaptozaobenzene (DMAB) on GNPs under light illumination. SERS can be used as a sensitive tool for monitor the above reaction and thus allow quantification of nitrite. Results related to the optimization of the analytical platform and the analyses of real samples are herein presented. The facile method could lead to further understanding of the progression and treatment of periodontitis and to guide professionals to effectively plan for on-site campaigns to control periodontal diseases.

## 2. Materials and Methods

### 2.1. Materials

Gold (III) chloride hydrate (HAuCl_4_), *p*-aminothiophenol (PATP), hexadecyltrimethylammonium bromide (CTAB), sodium citrate, hydroxylamine hydrochloride (NH_2_OH·HCl), phosphoric acid (85 wt.%), sodium nitrite (≥99.0%) were purchased from Sigma-Aldrich (Hangzhou, China). The above reagents were used without further purification and Milli-Q water (18 MΩ·cm^−1^) was used to prepare all aqueous solutions.

### 2.2. Preparation of GNP

Gold nanoparticles with different sizes were prepared to step by step previously described in references with slight modifications [31,32]. *GNP Seeds*: 5 mL of 1% trisodium citrate was quickly mixed with 1.7 mL of 1% HAuCl_4_ in 50 mL of boiling water solution under vigorous stirring; *GNP A*: 800 µL of 1% HAuCl_4_ was quickly adding to the solution containing 9.0 mL of 40 mM NH_2_OH·HCl, 10 mL of GNP seeds and 81 mL of H_2_O at room temperature. *GNP B*: 600 µL of 1% HAuCl_4_ was quickly adding to the solution containing 2.2 mL of 40 mM NH_2_OH·HCl, 40 mL of GNP A and 58 mL of H_2_O at room temperature; *GNP C*: 295 µL of 1% HAuCl_4_ was quickly adding to the solution containing 2.2 mL of 40 mM NH_2_OH·HCl, 25 mL of GNP B and 72 mL of H_2_O at room temperature; *GNP D*: 220 µL of 1% HAuCl_4_ was quickly adding to the solution containing 2.2 mL of 40 mM NH_2_OH·HCl, 20 mL of GNP C and 78 mL of H_2_O at room temperature; *GNP E*: 160 µL of 1% HAuCl_4_ was quickly adding to the solution containing 2.2 mL of 40 mM NH_2_OH·HCl, 40 mL of GNP D and 58 mL of H_2_O at room temperature.

### 2.3. Preparation of PATP-Prober

The obtained GNPs were further functionalized by PATP, as we previously described [33]. Briefly, 25 μL of 0.2 M CTAB and 50 μL of 1M HCl were mixed with 5 mL of the nanoparticles suspension under gentle stirring. After 10 min, an aliquot of 190 μL of 10 mM PATP (dissolved in 10% *v*/*v* ethanol) was added. The mixture was equilibrated at 60 °C for 2 h. Excess PATP in the mixture was removed by centrifugation and washing. The resulting PATP-capped GNPs (0.95 nM) were kept at 4 °C for use.

### 2.4. Saliva Nitrite Sensing

Experiments involving saliva from human volunteers were collected from volunteers. All subjects received informed consent forms before inclusion in the study and voluntarily agreed to participate. Samples of saliva were collected from the volunteers and were filtered using a 0.2 μm PTFE filter and adjusted to pH = 2 using 1M HCl. In a typical experiment, the obtained PATP-capped GNPs was firstly diluted to different particle concentration. Then, 20 μL of the diluted PATP-capped GNPs was mixed with NaNO_2_, resulting in different final concentrations of nitrite.

### 2.5. Instruments

The SERS spectra of the sensor were collected using a confocal Raman microscope system (LabRAM HR Evolution, Horiba Jobin Yvon, Paris, France), which was equipped with an integrated BX40 microscope (Olympus, Tokyo, Japan) and a motorized XYZ stage. High-resolution transmission electron microscopy (HRTEM: JEM-2010, JEOL Ltd., Tokyo, Japan, operated at 200 kV) and low-resolution transmission electron microscopy (TEM: H-800) were used to characterize the morphology microstructure. The UV–vis spectra were recorded using an Evolution 201 UV–vis spectrophotometer from Thermo Fisher (Waltham, MA, USA).

### 2.6. Numerical Simulation

FDTD simulations were performed using the commercial modeling package Lumerical solutions (Lumerical Solutions, Vancouver, BC, Canada). Periodic boundary conditions were employed, and only a GNP was placed in a unit cell. The optical constants of the gold were employed in the package.

## 3. Results

### 3.1. Sensing Principle

**Figure 1:**
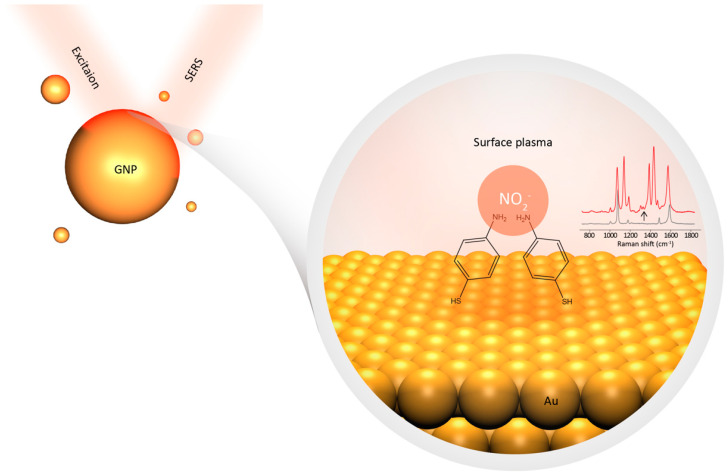
Schematic illustration of the sensing principle. NO_2_^−^ trigged surface plasmon-assisted conversion of PATP to DMAB on GNPs, resulting in a change in the SERS spectra of GNP.

Figure 1 schematically illustrates the sensing principle of this work, which is based on a nitrite-triggered SPAC reaction. NO_2_^−^ ions can trigger the rapid conversion of PATP to DMAB on GNPs under light illumination [33,34,35]. Such conversion can be easily in-situ witnessed through their SERS spectra. Three symbolic new bands at ca. 1140, 1390, 1432 cm^−1^ originating from DMAB can be seen as the proof of the above formation. The reaction mechanism of the conversion has been experimental and theoretically studied recently, in order to address the origin of these symbolic peaks. The relevant studies suggest that the NO_2_^−^ triggered conversation of PATP to DMAB on GNPs is a surface plasmon-assisted oxidation reaction, involving the transfer of “hot” electrons from PATP to NO_2_^−^ and protons, leading to the formation of DMAB [36,37,38]. The conversation is initiated by absorption of excitation energy (plasmonic heating) of the PATP on the GNPs, resulting in an electron loss of –NH_2_ group. The NO^2−^ ions serve as an oxidant and the electron acceptor. The key contributing factors of this complex reaction involve the nature of the SERS substrate, the excitation laser power, the pH value of reaction media, etc. However, the exact reaction mechanism is not clear.

Despite that, inspired by the high selectivity of the above NO_2_^−^ triggered catalysis reaction, a fast and straightforward nitrite screening method was firstly developed by our group [33] and used by other groups [37,39,40,41,42]. However, the detection limit of the proposed can be achieved about 10 μM. Meanwhile, to make the method fully suitable and robust for on-site fast screen or point-of-care application, this methodology needs further optimization, to understand the conversion of PATP to DMAB upon various physicochemical conditions.

### 3.2. Influence of the Particle Size of PATP-Prober

**Figure 2:**
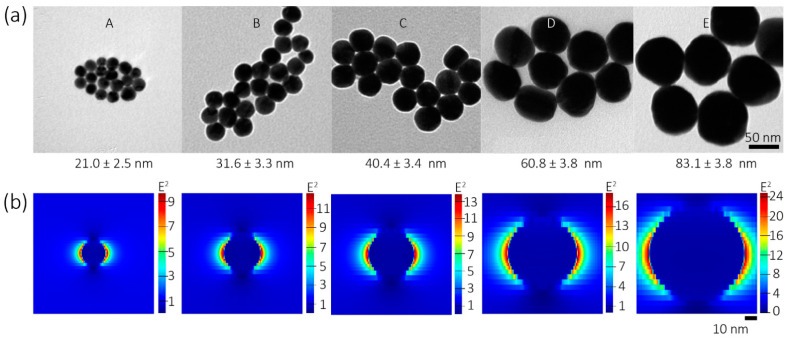

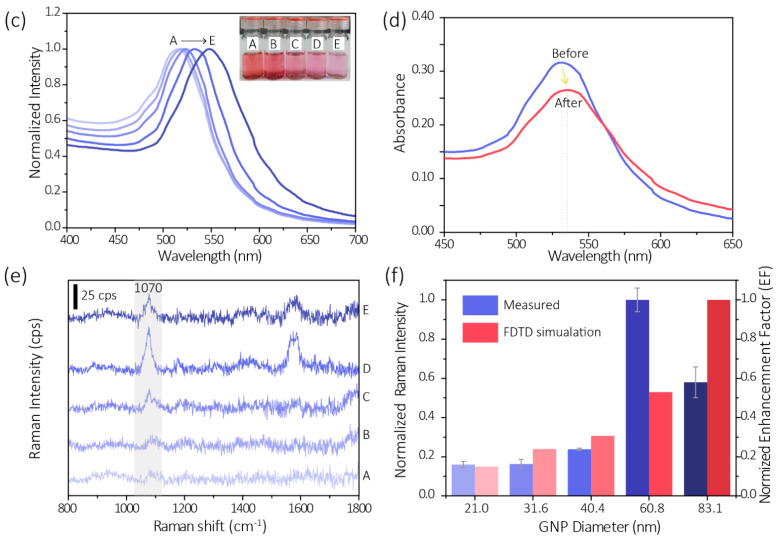
Influences of GNP size. (**a**) Typical TEM images of the GNP A–E used in this work were prepared by the seeding growth method. (**b**) FDTD simulations of the enhancement of field amplitude of the corresponding GNPs. In general, as the particle size increases, the amplitude increase. (**c**) LSPR absorption spectra of the obtained GNPs. The insert figure shows the photo of the obtained GNPs’ suspensions (GNP A–E). (**d**) Variation of LSPR absorption spectra before and after modification PATP. (**e**) SERS spectra of the obtained GNP-PATP probe with different particle sizes. (**f**) Comparison of the SERS intensities and situated SERS enhancement factor of different GNPs.

Surface plasmons give rise to the dramatic enhancement of electromagnetic (EM) fields near the surface of nanostructure. As a surface plasmon-assisted reaction, a key parameter affecting the sensitivity of this method is the morphology of GNP that can modulate the resulting surface plasmon, such as the sizes, shapes, etc. To study particle size’s influence on the sensitivity, we prepared different size GNPs using the seeding growth method described in the experimental section. Figure 2a shows the typical transmission electron microscopy (TEM) images of the obtained GNPs, which reveals the GNP were spherical and show excellent uniformity, with averaged diameters range from 20–80 nm. The details of these GNPs were summarized in Table 1.

To understand the size effect on surface plasmons, the 3D finite-difference time-domain (FDTD) method is used to simulate the surface plasmons (see the Materials and Methods Section). Figure 2b shows the enhancement of field amplitude of GNPs involved by the incident plane wave, indicating the enhancement of field amplitude increased significantly with the particle size. The LSPR yields exceptionally high absorption coefficients and scattering properties within the visible to near-infrared (NIR) wavelength range. As shown in Figure 2c, the redshift of LSPR peak was observed as the GNP increased; meanwhile, the color of the GNP solution is also affected significantly by the LSPR phenomenon. The damp of LSPR might due to the increase in the dielectric constant of the surrounding medium after the modification PATP.

We synthesized the PATP-GNP probe using the abovementioned GNPs. As shown in Figure 2d, GNP’s LSPR peak exhibited a slight red-shift after modification of GNP due to the increased refractive index changes induced by an PATP adsorbed on a GNP [33]. Notably, this result indicates that no aggregation of GNP-PATP occurred after the modification, for otherwise, a more wildly red-shift and new LSPR peak at 650–800 nm due to coupling to aggregated GNP would be seen. The obtained GNP-PATP remained well suspended in water. Figure 2e shows the corresponding SERS spectra of the obtained GNP-PATP (obtained using same particle concentrations), in which the three characteristic SERS bands at 1070, 1181, and 1581 cm^−1^ can be contributed to the mixed-mode from the C–C stretching and the C–S stretching, the C–H in-plane bending vibration, and the parallel C–C vibration stretching vibration, respectively [33].

Figure 2f shows measured SERS intensities of different GNP-PATP probes at 1070 cm^−1^. We noticed that the SERS intensities increased with the increasing GNP size between 21.0–60.8 nm, which is consistent with the enhancement factors obtained by FDTD simulation. However, the large PATP-GNP (80 nm) shows a large deviation from the FDTD result.

One possible reason could be that large GNPs are less stable than smaller particles after the modification with PATP. They tend to aggerate and are hard to redisperse after the centrifugal separation, which could cause the actual particle concentration of GNP to be less than the expected value. Therefore, larger PATP-GNP exhibited relatively weaker SERS intensity than expected. It should also be noticed that the FDTD simulation only considers the electromagnetic (EM) enhancement of GNP and neglects the chemical enhancement and other influences.

### 3.3. Influence of pH

**Figure 3:**
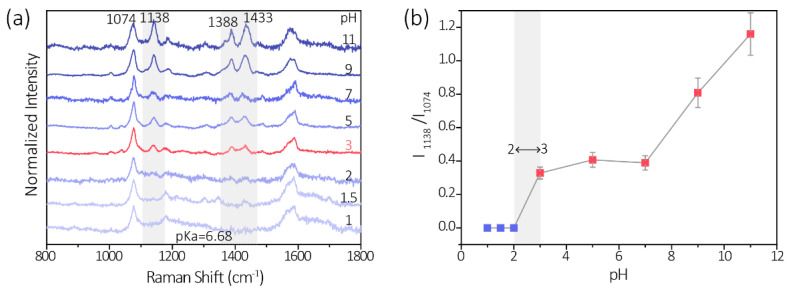
Influences of pH to the surface plasmon-assisted conversion of PATP to DMAB. (**a**) SERS spectra of the PATP-Au probe under various pH values (1–11). (**b**) Normalized Raman band intensities at 1138 cm^−1^ of PATP-Au probe under various pH values (1–11).

Accordingly, the 60 nm PATP-GNP was chosen for the following experiments since it exhibited a relatively strong signal and good stability. Another crucial parameter affecting the sensitivity of this method is the pH value of the solution. Figure 3a shows the pH-dependent SERS spectra of PATP-GNP probes. The results reveal that the conversion of PATP to DMAB on GNP automatically occurs at pH values ≥ 3, even in the absence of nitrite. We found that the three representative bands at ca. 1138, 1390, 1432 cm^−1^ originating from DMAB occurred without the addition of nitrite. (Figure 3a). When the pH was <3, we found that these three Raman peaks were absent, indicating the surface photocatalysis reaction could not happen. This phenomenon is more likely caused by the H^+^ and OH^−^ in the reaction media that can control surface photocatalysis reactions. In the case of a strong acidic environment, the PATP is protonated and the abundance of H^+^ prevents the oxidation of PATP to DMAB, also due to lack of electron acceptors in the media. However, under neutral and alkaline conditions, the absence of OH^−^ in the media may promote possible oxidation of PATP responsible for the appearance of *b*_2_ bands of the DMAB. These results are in agreement with the previously reported literature, and the possible reaction mechanism is proposed for controlling surface photocatalysis reaction by pH [37,43].

Figure 3b shows the corresponding Raman band intensities (1138 cm^−1^) of the PATP-prober under various pH values. Our finding indicates the PATP-prober is only stable in strong in a strongly acidic environment. Therefore, the above experimental results suggest the optimized pH value for our nitrite detection is pH ≤ 2.

### 3.4. Influence of Particle Concentration of PATP-Probe

**Figure 4:**
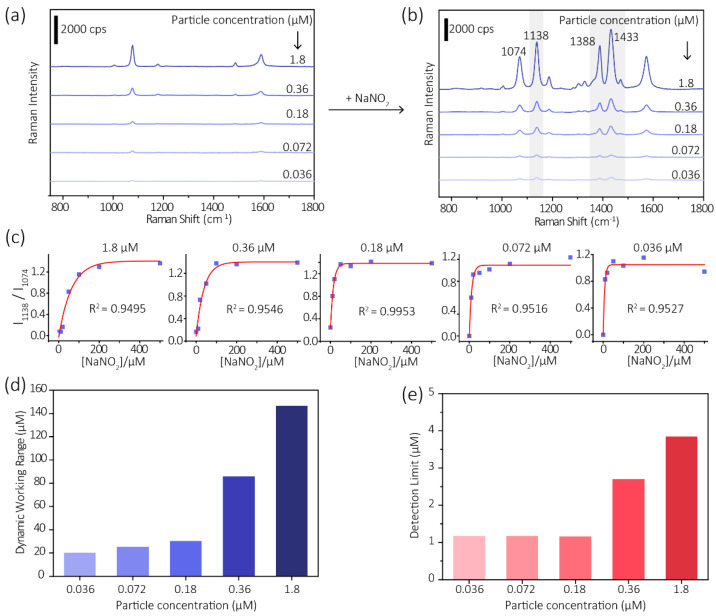
Influences of particle concentrations of GNP-PATP probe to the sensitivity of the assay. SERS spectra of GNP-PATP probe with different concentrations (**a**) before and (**b**) after addition of nitrite ions. (**c**) Normalized Raman peak intensity at ~1141 cm^−1^ of the GNP-PATP probe to various nitrite concentrations, and the resulting (**d**) dynamic working range and (**e**) detection limits using different particle concentrations of GNP-PATP.

To investigate the particle concentration’s influence on the sensitivity, we tested the results using different particle concentrations. As shown in Figure 4a,b, with nitrite and PATP-GNP with varying particle concentrations, conversion happens. 

Figure 4c shows the probe response to various nitrite concentrations. Normalized Raman peak intensity at ~1141 cm^−1^ is shown (normalized to the peak intensity at ~1070 cm^−1^), which increased with the increase of nitrite concentration initially in a relatively low range, and become saturated at high concentration (200–500 μM). We noticed that the sensor’s dynamic working range is directly related to the particle concentration. As the particle concentration decreases the dynamic working range narrows, which is reasonable due to the drop of probe number in the system. 

The limit of detection (LOD) of this method is estimated according to the 3σ rule. We noticed that the LOD also improved with the decrease of particle number due to the lower baseline value. Under the optimized conditions, we can achieve a 1 μM LOD, which is ten times lower than in our previous study.

### 3.5. POC Application

**Figure 5:**
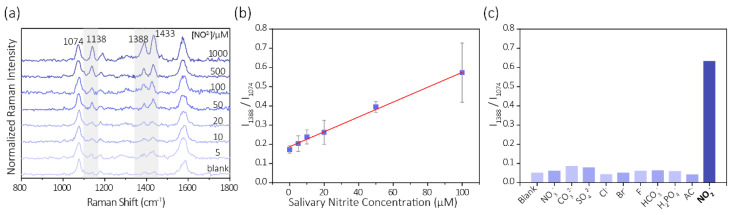
Quantification of nitrite in human saliva. (**a**) SERS spectra of GNP-PATP probe to the saliva sample containing various nitrite. with different concentrations. (**b**) Calibration curves are generated by plotting the normalized intensity of the peaks at 1388/1074 cm^−1^ as a nitrite concentration function. (**c**) Normalized intensities of the peaks at 1388/1074 cm^−1^ towards competing ions (Competing ions and NO_2_^−^: 100 µM).

Under the optimized experimental conditions, the quantification of nitrite in saliva was based on a standard external method utilizing the calibration relationship between normalized peak heights vs. the concentration of nitrite standards. Figure 5a shows the characteristic Raman bands at ca. 1138, 1388, 1433 cm^−1^ for different concentrations of nitrite over the range of 0 to 1000 µM. All spectra were baseline-corrected and then normalized with respect to the internal standard Raman intensity at ca. 1074 cm^−1^ that is mainly due to the C-S stretching.

This band is thought to remain largely unchanged during the detection. As shown in Figure 5b, we also noticed there existed a linear relationship between the normalized SERS intensity (*I*_1388_/*I*_1074_) in the spectra and nitrite concentrations. The calibration curves of eight concentrations showed an excellent linear response in the range of nitrite 1–100 µM, with an R^2^ of 0.9579. The detection limit was 1 µM (0.046 ppm), which is estimated according to the *3σ* rule. 

The LOD we achieved in this work is ten times lower than in our previous study, which is also well below the salivary nitrate levels that were found in infants (~1 ppm) and adults (~7 ppm) [44]. The selectivity of this nitrite sensing scheme was verified in Figure 5c, and was thoroughly studied in our previous work [33] and many other previous works [37,39,42].

## 4. Discussion

Nitrite commonly exists in food and agricultural products, as a result of its wide usage as food additive or in chemical fertilizers [1]. However, its possible health threats have raised the alarm for close inspection of the nitrite level in food and agricultural products. On other hand, the NO^2−^ can also be used as a health biomarker for the human body. The nitrite levels in body fluids such as saliva and urine are found to be closely related to certain diseases [5,6,7]. Salivary nitrite is reduced by oral bacteria associated with the clinical symptoms of periodontitis (gingival redness, swelling, and bleeding) and can be used to trace oral diseases such as periodontitis. Such diseases can lead to the formation of tooth abscess, tooth loss, or infection of the surrounding tissue and bones and increase the risk factors for systemic diseases such as coronary heart disease, atherosclerosis, and stroke. Therefore, rapid on-situ detection of NO^2−^ is of great necessity for both food safety and medical diagnosis [45].

Although conventional nitrite analytical methods can achieve sound selectivity and sensitivity [8,9,11,12,13], they suffer some unavoidable limitations such as the need for sophisticated pretreatment procedures, being time-consuming and high cost, and the requirement for professional operation, which limit their applications in rapid on-site detection and point-of-care (POA) tests. Therefore, it is necessary to develop a rapid, inexpensive, and pretreatment-free analytical approach for nitrite sensing.

Inspired by a nitrite-triggered SPAC reaction, we developed a nitrite screening method in our previous work [33]. The proposed method is simple, pretreatment-free, and fast (detection time <1 min), which is highly desirable for rapid on-site detection and point-of-care (POA) tests. However, the LOD it can achieve is about 10 μM, which just meets the proposed EPA limit for nitrite in drinking water (21.7 µM). Lately, the similar sensing scheme was adopted by many groups to detect nitrite, but using more complex SERS-active nanoparticles (such as Ag Nanopyraid, Au nanostar, Fe_3_O_4_/Au, Fe_3_O_4_/Au@Ag, etc.) to improve the sensitivity [37,39,40,41,42], which could also increase the time and difficulties for preparing the sensing probers. Other approaches to improve the sensitivities include by adding an additional reactant (1-naphthylamine) to boost the signal (the Griess reaction) [46,47,48,49]. However, in this work, we have shown an alternative approach, that by simply optimizing GNP size, particle concentrations, pH value of the reaction media, the LOD can be improved to 1 μM, which is ten times more sensitive than our previous method.

One limitation of our method is the readout system. Currently, a large Raman spectrometer is needed for signal readout, which is unsuitable for POC testing, but a feasible solution would be to develop a portable and inexpensive Raman spectrometer that allows patients to check saliva nitrite levels outside the lab.

In conclusion, we have reported a new method for nitrite quantification in saliva based on a nitrite-triggered surface plasmon-assisted catalysis (SPAC) reaction. In this work, we optimized many essential parameters for this method, such as GNP size, particle concentration, and pH. The proposed method exhibits good sensitivity and a rapid response. These promising results suggest its great potential as an on-site fast screen or point-of-care testing method.

## Figures and Tables

**Table 1:** Summary of GNPs. The number concentration of GNP seeds (2.5 × 10^−8^ M) was determined according to Beer’s law by using the extinction coefficient of 10^8^ M^−1^·cm^−1^ for 13 nm GNP in diameter at 520 nm. According to previous reports [33], duction of Au^3+^ by NH_2_OH is dramatically accelerated by Au surfaces, as a result, no new particle nucleation occurs during the preparation of GNP A to GNP E. Therefore, their number concentration can be calculated based on the smaller GNP were added as seeds. The NP sizes were verified by TEM images (at least 200 particles were measured, respectively).

	GNP A	GNP B	GNP C	GNP D	GNP E
λ_max_/nm		519	522	533	549
Extinction/cm^−1^	0.6251	0.6295	0.3675	0.3216	0.2563
Particle concentration/µM	18	7.2	1.8	0.36	0.144
Diameter/nm	21.0 ± 2.5	31.6 ± 3.3	40.4 ± 3.4	60.8 ± 3.8	83.1 ± 4.4

## Data Availability

All data needed to evaluate the conclusions in the paper are present in the paper.
